# Development of caesarean section prediction models: secondary analysis of a prospective cohort study in two sub-Saharan African countries

**DOI:** 10.1186/s12978-019-0832-4

**Published:** 2019-11-14

**Authors:** Hayala C. C. de Souza, Gleici S. C. Perdoná, Alessandra C. Marcolin, Lawal O. Oyeneyin, Olufemi T. Oladapo, Kidza Mugerwa, João Paulo Souza

**Affiliations:** 1 Department of Social Medicine, Ribeirão Preto Medical School, University of São Paulo, Av. Bandeirantes, 3900 - Vila Monte Alegre, Ribeirão Preto -, Ribeirão Preto, SP Brazil; 2 Department of Gynecology and Obstetrics, Ribeirão Preto Medical School, University of São Paulo, Av. Bandeirantes, 3900 - Vila Monte Alegre, Ribeirão Preto -, Ribeirão Preto, SP Brazil; 3 Department of Obstetrics & Gynaecology, University of Medical Sciences Teaching Hospital, Medical, village, Laje road, Ondo City, Ondo State Nigeria; 4 UNDP/UNFPA/UNICEF/WHO/World Bank Special Programme of Research, Development and Research Training in Human Reproduction (HRP), Department of Reproductive Health and Research, World Health Organization, Avenue Appia 20, 27, CH-1211 Geneva, Switzerland; 5 Department of Obstetrics and Gynaecology, Makerere University, Kampala, Uganda

**Keywords:** Caesarean section, Logistic regression, Prediction models, Decision support models

## Abstract

**Background:**

Caesarean section is recommended in situations in which vaginal birth presents a greater likelihood of adverse maternal or perinatal outcomes than normal. However, it is associated with a higher risk of complications, especially when performed without a clear medical indication. Since labour attendants have no standardised clinical method to assist in this decision, statistical tools developed based on multiple labour variables may be an alternative. The objective of this paper was to develop and evaluate the accuracy of models for caesarean section prediction using maternal and foetal characteristics collected at admission and through labour.

**Method:**

This is a secondary analysis of the World Health Organization’s Better Outcomes in Labour Difficulty prospective cohort study in two sub-Saharan African countries. Data were collected from women admitted for labour and childbirth in 13 hospitals in Nigeria as well as Uganda between 2014 and 2015. We applied logistic regression to develop different models to predict caesarean section, based on the time when intrapartum assessment was made. To evaluate discriminatory capacity of the various models, we calculated: area under the curve, diagnostic accuracy, positive predictive value, negative predictive value, sensitivity and specificity.

**Results:**

A total of 8957 pregnant women with 12.67% of caesarean births were used for model development. The model based on labour admission characteristics showed an area under the curve of 78.70%, sensitivity of 63.20%, specificity of 78.68% and accuracy of 76.62%. On the other hand, the models that applied intrapartum assessments performed better, with an area under the curve of 93.66%, sensitivity of 80.12%, specificity of 89.26% and accuracy of 88.03%.

**Conclusion:**

It is possible to predict the likelihood of intrapartum caesarean section with high accuracy based on labour characteristics and events. However, the accuracy of this prediction is considerably higher when based on information obtained throughout the course of labour.

## Plain English summary

Caesarean section (CS) is a surgical procedure indicated in situations in which vaginal delivery presents a higher likelihood of adverse maternal and/or perinatal outcomes than normal. In principle, it should not be performed without a clear medical indication, i.e., a clear likelihood that it will improve outcomes. However, the CS rates have continuously increased, which is a worrying scenario since CS can lead to complications as well. The situation is worse when CS are performed unnecessarily because then, it presents no clear benefit and only potential damages. To improve this scenario, it is important that attendants have tools that support the decision toward the most appropriate mode of birth. Statistical models can be used as part of these tools. Therefore, the aim of this study was to develop statistical models for CS prediction based on maternal and foetal characteristics. We considered admission and labour data from 8957 pregnant women from two African countries (Nigeria and Uganda). We developed a set of prediction models, which showed high performance on predicting CS, mainly when the labour characteristics are considered. Based on these results, we believe these models can be useful adjunct tools in helping medical decision-making.

## Background

Since its introduction in clinical practice, the CS rates have continuously increased across the world [1, 2]. Recent estimates suggest that the CS rates have doubled between 2000 and 2015 in some regions of the world [3]. This trend is hardly justified, since CS rates greater than 10% have not been associated with the reduction of maternal and perinatal mortality [4, 5]. In addition, elective CS is associated with higher rates of mortality and complications in the short and long term [6–10]. These risks are greatest in countries with low resources and high fertility rates, such as those in sub-Saharan Africa [11–13].

Early identification and appropriate management of women at risk of CS may improve outcomes [14]. However, labour attending teams have no standardized method to help to decide when CS is most appropriate in situations when the medical indication is not clear-cut [15, 16]. Sociodemographic, gestational, peri-partal and cultural factors may influence the choice of mode of childbirth and vary according to the development levels of each country [11, 16]. Therefore, to improve labour outcomes, it is important to develop tools based on multiple variables that contribute to decision-making processes before the woman reaches an increased risk of complications. Proper use of these tools could avoid unnecessary CS and emergency surgeries, reduce rates of severe maternal and perinatal outcomes.

In order to meet these objectives, some researchers have proposed statistical models that take into account the baseline and admission characteristics in the labour of a pregnant woman and the foetus [17–24]. Most of these models considered data from only one country such as Canada [22], the United States [18, 21], Scotland [23], Ireland [17], England [19] and Spain [20]. We identified only one CS prediction model developed with information of pregnant women from several countries including both low- and high-income settings; however, this model also used static information collected at labour room admission [24]. Harper and colleagues proposed a prediction model considering intrapartum information [25] from deliveries at a University Medical Centre in United States. As far as we know, few researches present models built with low-resource country data using labour related characteristics. Considering potential differences in the characteristics of the population and the response capacity of health facilities (among other factors), it would be advisable the development of such models based on information generated in low-resource settings in order to reduce indirectness and improve relevance and applicability of the tools.

In this article, we consider the hypothesis that statistical models can predict the occurrence of CS and that this prediction may be more accurate using intrapartum variables. Therefore, our objective was to develop CS prediction models and evaluate their accuracy in two sub-Saharan African countries.

## Methods

We conducted a secondary analysis of the database from the Better Outcomes in Labour Difficulty (BOLD) project, a World Health Organization’s multicentre study aimed at accelerating the reduction of maternal, foetal and neonatal mortality and morbidity related to intrapartum. The researchers collected this database from the prospective cohort performed as part of the BOLD project. A methods paper presents a detailed description of the project study protocol [26]. In summary, BOLD researchers collected data from women admitted for labour care in 9 and 4 hospitals in Nigeria and Uganda, respectively, from 2014 to 2015. To be selected, hospitals must have a minimum of 1000 births per year, be the major health care facility in its region, and not a primary care unit. Intrapartum care was provided by skilled birth attendants, with stable access to CS, augmentation of labour, assisted vaginal delivery and good obstetric care practices [27, 28].

BOLD cohort considered eligible women admitted for spontaneous or induced vaginal delivery, with a single foetus, during the first stage of labour (both in the latent phase and in the active phase), with cervical dilatation less than 7 cm. The following women were excluded: pregnant women diagnosed with foetal death, cervical dilatation ≥7 cm, multiple gestation, gestational age less than 34 weeks, elective or pre-labour CS, with an indication of emergency CS or laparotomy on admission, failed induction of labour, false labour, unemancipated minors without a legal guardian, and women who were not able to give consent. Trained nurses carried out the recruitment process.

The main outcome of the present analysis was the occurrence of CS and the predictors are the maternal characteristics of admission and intrapartum variables evaluated in the first and second stages of labour. As the CS can be objectively measured, we reduce the potential detection bias in the context of a multicentre study. The BOLD project also recorded the dates and times of the interventions performed, as well as maternal and perinatal outcomes. The research team used a standardized collection form developed for the BOLD project and they collected the records during childbirth according to routine obstetric care protocols of the hospitals. They calculated the sample size based on the set of maternal and perinatal outcomes defined in the BOLD and considered 20 possible predictors. Based on initial assumptions, a minimum required sample size of 7812 women was calculated. The BOLD research team was concerned with avoiding potential biases throughout the development of the project, in steps such as: choosing the study design, developing the data collection instrument and managing the data to ensure its quality [26].

To describe the demographic characteristics of the women in the study, we present mean and standard deviation of quantitative variables and percentage and absolute frequencies for qualitative variables. For the study of the main hypothesis, we developed logistic regression models in which the dependent variable was CS, and the independent variables were baseline (fixed) and intrapartum (dynamic) measurements of the pregnant women. The logistic regression equation based in one independent variable is given by

$$ CSProbability=\frac{e^{\beta_0+{\beta}_1{X}_1}}{1+{e}^{\beta_0+{\beta}_1{X}_1}}, $$

where *β*_0_ is the equation intercept and *β*_1_ is the coefficient related to the independent variable *X*_1_ [29]. To calculate the probability of CS for a specific woman, when the independent variable is continuous, simply replace *X*_1_ with the value observed for that woman. When the independent variable is categorical, *X*_1_ represents the presence of a characteristic and must be replaced by 1 or 0 if the characteristic is present or absent, respectively. If the prediction model has more than one independent variable, the equation is analogous:

$$ CSProbability=\frac{e^{\beta_0+{\beta}_1{X}_1+{\beta}_2{X}_2+{\beta}_3{X}_3+\dots }}{1+{e}^{\beta_0+{\beta}_1{X}_1+{\beta}_2{X}_2+{\beta}_3{X}_3+\dots }}. $$

We present three types of models, which differ from one another in terms of the moment of recording the intrapartum variables used: the labour admission model, the interval models and the maximum score model. To obtain the labour admission model, we first selected variables that presented *p*-values lower than 5% in the chi-square and Student t-tests in the complete sample (bivariate analysis). We considered the admission records of the following variables in bivariate analysis:

- Fixed: Maternal height, Symphysis-fundal height, Foot size (mother), BMI, Parity and previous caesarean section (CS), Previous uterine surgery, Previous abortions, History of prolonged labour, Outcome of last pregnancy, Complications with current pregnancy, Chronic health conditions prior to pregnancy, Gestational age, Mode of onset of labour, Cervix position.
- Dynamic: Cervical dilatation, Maternal heart rate (MHR), Systolic blood pressure (SBP), Diastolic blood pressure (DBP), Number of uterine contractions in 10 min, Suspected foetal distress, Foetal movements, Foetal station, Foetal presentation, Amniotic membrane status, Abnormal axillary temperature, Moderate to extreme pain, *Moulding* status*, Caput succedaneum.*

We categorized the following quantitative predictors: Maternal BMI, SBP, DBP, MHR, Axillary temperature, Number of uterine contractions in 10 min. For Maternal BMI, we considered the categorization according to the gestational age [30]. The MHR and blood pressure were categorized according to the Modified Early Obstetric Warning Score [31]. We used the foetal heart rate (FHR) values to define the suspected foetal distress variable (i.e. values above 160 beats or below 120 beats were considered as suspected foetal distress [32]). We considered as abnormal axillary temperature, values below 35.5 °C and equal or greater than 37.5 °C. Additional file 1: Table S1 and Additional file 1: Table S2, respectively, present more details about the fixed and dynamic variables considered in the bivariate analysis step. The final admission model (Model 1) included those independent variables selected using a stepwise selection method among the significant variables in bivariate analysis. The stepwise is a method that sequentially adds variables into the model that most improves the fit [33]. To evaluate the model improvement at each step, we considered de Akaike information criterion [34].

Interval models include the same predictors of final admission model, however, we considered updated values for the last measured measure of the dynamic variables, at three time intervals, from 4 cm of dilatation: 0 to 2 h (Model 2), 2 to 4 h (Model 3) and 4 to 6 h (Model 4). In these models, the elapsed time between admission and the first record of dilatation equal to 4 cm was inserted as an additional independent variable. For the interval models, only women with a record of a 4 cm dilatation were included.

To obtain the maximum score model (Model 5), we considered the same fixed variables from final admission model. Regarding dynamic variables, we used bivariate analysis to select records related to conditions presented throughout the labour among the described below:

- Final cervical dilatation, Most extreme MHR, Most extreme SBP, Most extreme DBP, Highest number of uterine contractions observed (10 min), At least one suspected foetal distress, Presence of foetal movements throughout intrapartum, Lower foetal station achieved, Final foetal presentation, Final amniotic membrane status, Some occurrence of abnormal axillary temperature, Some occurrence of moderate to extreme pain, Highest *Moulding* status*,* Most severe level of *Caput succedaneum.*

**Fig. 1:**
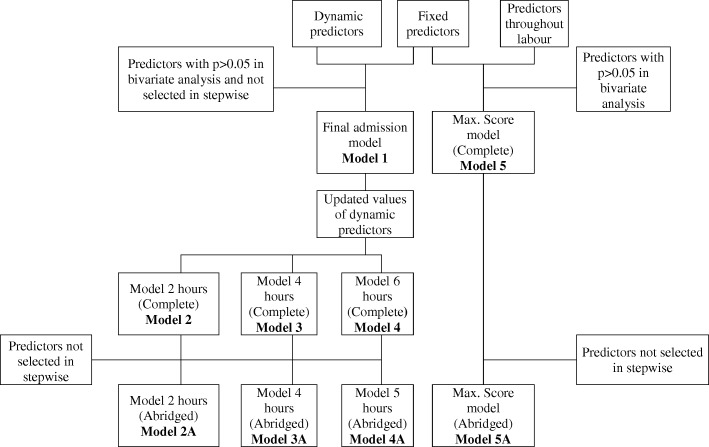
Schematic flow diagram of model building

Additional file 1: Table S3 presents more details about the variables listed above. We also present an abridged version of each interval model and of maximum score model, that was obtained using a stepwise selection method. Figure 1 presents a schematic flow diagram including a summary of the models studied. In summary, Model 1 returns a probability of CS at admission, Models 2 to 4 return the probability of CS at 2, 4 and 6 hours after the start of the active phase and Model 5 returns the probability of CS throughout the labour.

For the model estimation, we used random samples containing 70% of the data set that did not present missing data. This segment was called the training sample, while the remaining 30% (test sample) was used in the validation step. It is important to divide the dataset, because it allows us to evaluate the model discriminatory capacity using a sample that was not the same used to fit the model.

To evaluate the discriminatory capacity, we present the ROC Curve and the area under the ROC curve (AUC). The ROC curve graphically represents the values of sensitivity and specificity for an entire range of prediction cut-off points. Sensitivity reflects the proportion of individuals for whom the model correctly indicated by performing CS among all those who actually undergone to CS. Specificity is the proportion of individuals that the model indicates for not performing CS among all those who actually did not. The higher the discriminatory capacity of a model, the higher its AUC, which can reach a maximum of 1 (or 100%).

We also presented the diagnostic accuracy (DA), positive predictive value (PPV) and negative predictive value (NPV) related to the cut-off point that maximizes sensibility and specificity. DA is the proportion of individuals in which the model is correct among all individuals. PPV and NPV are the proportion of observations in which the model is correct among that one the model indicates to perform and to not perform the CS, respectively. To evaluate the adequacy of fitted models to the data, we used the Hosmer-Lemeshow test, which evaluates the null hypothesis that the logistic model is the correct choice. The analyses were performed using the R software version 3.5.1 [35].

## Results

There were 9995 women in the database, out of which 8957 women without any inconsistency in the time of intrapartum record were used. Thirty percent of women (*n* = 2684) had at least one record taken at 4 cm of dilatation. From these, 2683 have at least one record between 0 and 2 h after dilatation of 4 cm, 1604, between 2 and 4 h and 714, between 4 and 6 h. Additional file 1: Figure S1 presents the analysis flow diagram. Table 1 presents the description of the sociodemographic data and the outcomes. The CS represented 12.7% of the deliveries performed. Among all women who had CS, the main indications were: suspected acute foetal distress (31.3%), cephalopelvic disproportion (16.3%) and prolonged labour (15.7%).

**Table 1:** Socio-demographic data of outcomes for the study population from the BOLD project

	Uganda (*N* = 4302)		Nigeria(*N* = 4655)	
	Mean	SD	Mean	SD
Age	26.87	4.98	28.94	4.85
	*N*	% (95% CI)	*N*	% (95% CI)
Marital status				
No partner	157	3.7(3.1;4.3)	45	1(0.7;1.3)
Partner	4143	96.3(95.7;96.9)	4609	99(98.7;99.3)
Education level				
None/ Pre-primary/Other	144	3.4(2.9;4)	97	2.1(1.7;2.6)
Complete/incomplete primary	515	12.1(11.1;13.1)	249	5.4(4.8;6.1)
Complete/incomplete secondary	2023	47.4(45.9;48.9)	1732	37.5(36.1;38.9)
Post-secondary/Tertiary complete/incomplete	1584	37.1(35.7;38.6)	2540	55(53.6;56.4)
Outcomes				
Caesareans sections	634	14.7(13.7;15.8)	509	10.9(10.1;11.9)
Livebirths	4298	99.9(99.8;100)	4639	99.7(99.4;99.8)
Stillbirths	4	0.1(0;0.2)	16	0.3(0.2;0.6)
Still births + neonatal deaths occurred during the first day of life	20	0.5(0.3;0.7)	26	0.6(0.4;0.8)
Maternal death	11	0.3(0.1;0.5)	8	0.2(0.1;0.3)
Maternal near-miss	5	0.1(0;0.3)	5	0.1(0;0.3)

Table 2 presents the performance measures for all the estimated models and Additional file 1: Figure S2, the ROC curve of each of the models when applied to the training and test samples. Table 3 presents the estimated coefficients for the admission model and for the complete and abridged interval models and Table 4 presents the coefficients estimated for the maximum score models. From a practical point of view, positive coefficients indicate that this variable increases the probability of performing CS. On the other hand, negative coefficients indicate that a certain variable reduces the probability of CS.

**Table 2:** Performance measures for the complete and abridged models in the training and test samples

		Cut-off point	SP	SE	DA	NPV	PPV	AUC	AUC CI (95%)	
Training sample										
Admission	Model 1	16.12	78.72	63.84	76.84	93.79	30.18	77.76	76.01	79.50
2 h	Model 2	18.91	71.42	74.43	72.00	92.04	38.61	78.76	75.98	81.54
	Model 2A	18.96	66.43	76.44	68.59	91.10	38.55	78.07	75.44	80.70
4 h	Model 3	21.76	77.83	76.44	77.53	92.45	48.19	85.92	83.03	88.81
	Model 3A	32.02	85.04	71.28	82.04	91.40	57.02	85.65	82.86	88.44
6 h	Model 4	23.57	77.78	79.75	78.26	92.20	53.85	85.14	80.46	89.83
	Model 4A	35.96	86.40	62.82	80.79	88.16	59.04	84.31	79.60	89.02
Max. score	Model 5	13.66	87.90	85.20	87.56	97.61	50.60	93.80	92.88	94.71
	Model 5A	10.69	84.69	87.96	85.12	97.91	46.30	93.66	92.77	94.54
Test sample										
Admission	Model 1	16.12	78.68	63.20	76.62	93.30	31.28	78.70	76.19	81.20
2 h	Model 2	18.91	70.15	67.65	69.64	89.52	36.51	75.88	71.50	80.27
	Model 2A	18.96	66.38	68.42	66.71	91.41	28.68	74.73	69.74	79.72
4 h	Model 3	21.76	77.17	76.00	76.92	92.21	47.50	85.29	80.79	89.78
	Model 3A	32.02	88.47	64.00	83.51	90.63	58.54	84.39	79.43	89.36
6 h	Model 4	23.57	76.58	77.78	76.81	93.41	44.68	85.19	77.42	92.95
	Model 4A	35.96	82.88	70.00	80.14	91.09	52.50	86.52	80.07	92.96
Max. score	Model 5	13.66	89.26	80.12	88.03	96.66	53.68	93.66	92.36	94.96
	Model 5A	10.69	84.69	86.81	84.95	97.81	44.92	93.72	92.33	95.11

*SP: Specificity, SE: Sensitivity, DA: diagnostic accuracy, NPV: negative predictive value, PPV: positive predictive value, AUC: area under the ROC curve, CI: Confidence interval (CI)

**Table 3:** Coefficients of admission and interval models to predict CS

	ADM	2 h		4 h		6 h	
	Model 1	Model 2	Model 2A	Model 3	Model 3A	Model 4	Model 4A
	(*N* = 5908)	(*N* = 1568)	(*N* = 1611)	(*N* = 819)	(*N* = 863)	(*N* = 322)	(*N* = 328)
Intercept	−4.611	−5.894	−5.326	−0.113	0.724	−9.510	−4.637
Symphysis-fundal height	0.176	0.130	0.134	0.043		0.145	0.123
Dilatation	−0.158	− 0.232	− 0.257	− 0.689	−0.686	− 0.403	−0.494
Age	0.002	0.005		0.036	0.035	0.060	0.020
Maternal height	−0.020	0.001		−0.005		0.015	
BMI: Normal	–	–	–	–	–	–	
BMI: Underweight	−0.150	− 0.437	− 0.469	−0.399	− 0.683	−0.263	
BMI: Overweight	0.239	0.421	0.421	0.211	0.249	0.365	
BMI: Obese	0.337	0.302	0.464	0.458	0.577	0.433	
Mode of onset of labour: Spontaneous	–	–		–		–	
Mode of onset of labour: Induced	0.039	0.183		0.262		−0.436	
Parity: Nulliparous	–	–	–	–	–	–	–
Party: Multiparous without CS	− 1.275	− 1.086	−1.184	− 1.160	− 1.360	− 1.270	− 0.832
Parity: Multiparous with CS	0.868	0.729	0.905	0.138	0.425	1.077	1.637
Ischial spines prominent: No	–	–	–	–	–		
Ischial spines prominent: Yes	1.107	1.762	2.150	1.519	1.057		
Cervix position: Anterior	–	–	–	–		–	
Cervix position: Central	−0.082	0.346	0.197	−0.066		− 0.207	
Cervix position: Posterior	0.308	0.602	0.442	0.025		0.134	
Gestational age: Term	–	–		–	–	–	
Gestational age: Pre-term	−0.214	−0.361		− 0.682	−1.136	0.048	
Gestational age: Post-term	0.329	0.161		1.008	0.660	−0.007	
Foetal presentation: Cephalic anterior	–						
Foetal presentation: Cephalic transverse	1.139						
Foetal presentation: Cephalic posterior	0.889						
Foetal presentation: Other	0.688						
MHR: 50–100	–	–		–	–	–	–
MHR: 40–49 or 101–110	0.156	0.041		1.441	0.914	1.377	1.427
MHR: < 40 or > 110	0.560	0.728		0.248	0.378	1.377	1.427
SBP: 90–139	–	–		–		–	–
SBP: 80–89 or 140–149	−0.020	0.253	0.078	0.441		−1.190	−0.106
SBP: 71–79 or 150–159	0.356	0.321	0.603	−0.340		1.928	1.241
SBP: <=70 or > =160	0.146	1.172	0.840	−1.029		1.157	0.835
Number of contractions observed (10 min) > =3	–	–		–	–	–	–
Number of contractions observed (10 min) < 3	0.555	0.009		0.362	0.414	0.839	0.456
Suspected foetal distress: No	–	–	–	–	–	–	–
Suspected foetal distress: Yes	0.657	1.207	0.790	2.509	2.121	1.683	1.187
Foetal movements: No	–	–	–	–		–	
Foetal movements: Yes	−0.405	−0.431	−0.388	0.046		0.233	
Foetal station: Above the ischial spines	–	–	–	–		–	
Foetal station: At or below the ischial spines	− 0.535	− 0.716	− 0.441	− 0.160		−0.420	
Amniotic membrane: intact	–	–	–	–	–	–	–
Amniotic membrane: ruptured with meconium	0.758	0.513	0.500	0.617	0.658	1.189	1.492
Amniotic membrane: ruptured without meconium	−0.041	1.589	1.537	1.336	1.321	1.978	2.413
Labour pain: No	–	–		–	–	–	
Labour pain: Moderate to extreme	−0.178	0.118		0.583	0.447	0.346	
Time between admission and 4 cm: < 8 h		–	–	–		–	–
Time between admission and 4 cm: > = 8 h		0.433	0.410	− 0.321		0.668	0.529

BMI: Body Mass Index; MHR: Maternal Heart Rate; SBP: Systolic Blood Pressure;

**Table 4:** Coefficients and p-values of complete and abridged version of maximum score models to predict CS

	Model 5 (*N* = 5850)		Model 5R (*N* = 6048)	
	Coef.	*p*-value	Coef.	*p*-value
Intercept	−9.0707	0.001	−6.9977	0.001
Symphysis-fundal height (cm)	0.2029	0.001	0.1841	0.001
Final cervical dilatation	−0.5602	0.001	−0.5594	0.001
Age	0.0158	0.237		
Maternal height	0.0066	0.478		
BMI: Normal	–			
BMI: Underweight	0.0036	0.984		
BMI: Overweight	−0.0120	0.934		
BMI: Obese	0.0574	0.732		
Mode of onset of labour: Spontaneous	–		–	
Mode of onset of labour: Induced	0.2226	0.198	0.2888	0.075
Parity: Nulliparous	–		–	
Parity: Multiparous without previous CS	−1.3044	0.001	−1.1991	0.001
Parity: Multiparous with CS	0.9186	0.001	0.9867	0.001
Ischial Prominent spines: No	–		–	
Ischial Prominent sciatic spines: Yes	1.2837	0.004	1.0130	0.029
Cervix position: Anterior	–		–	
Cervix position: Central	−0.0885	0.544	0.0071	0.960
Cervix position: Posterior	0.4972	0.008	0.4373	0.019
Gestational age: Term	–		–	
Gestational age: Pre-term	−0.4344	0.089	−0.3899	0.098
Gestational age: Post-term	0.5000	0.109	0.4545	0.110
Final foetal presentation: Anterior cephalic	**–**		**–**	
Final foetal presentation: transverse cephalic	0.5579	0.361	0.2786	0.640
Final foetal presentation: posterior cephalic	2.3656	0.001	2.4717	0.001
Final foetal presentation: Others	1.6676	0.001	1.7075	0.001
Most extreme MHR: 50–100	–			
Most extreme MHR: 40–49 or 101–110	0.1205	0.568		
Most extreme MHR: < 40 or > 110	0.3007	0.323		
Most extreme SBP: 90–139	–			
Most extreme SBP: 80–89 or 140–149	0.2456	0.190		
Most extreme SBP: 71–79 or 150–159	0.1383	0.606		
SBP: <=70 or > =160 (Risk 3)	0.2512	0.301		
Highest number of contractions observed (10 min) > =3	–		–	
Highest number of contractions observed (10 min) < 3	0.9000	0.001	1.0098	0.001
At least one suspected foetal distress: No	–		–	
At least one suspected foetal distress: Yes	2.3763	0.001	2.5335	0.001
Foetal movements: At least an absence of foetal movements	**–**		–	
Foetal movements: present throughout intrapartum	−0.3616	0.003	−0.3275	0.005
Lower foetal station achieved: Above ischial spines	**–**		–	
Lower foetal station achieved: At or below ischial spines	−1.0640	0.001	−0.9810	0.001
Final amniotic membrane: Intact	**–**		–	
Final amniotic membrane: rotated with meconium	2.7469	0.001	2.9515	0.001
Amniotic membrane: rotated without meconium	1.1715	0.001	1.5163	0.001
Labour pain: No occurrence of moderate to extreme pain	**–**		–	
Labour pain: Some occurrence of moderate to extreme pain	0.7488	0.001	0.5317	0.001
Axillary temperature: No occurrence of abnormal axillary temperature	**–**		–	
Axillary temperature: Some occurrence of abnormal axillary temperature (< 36 or > 37.4)	0.4553	0.005	0.1881	0.240
Highest *Moulding:* None	**–**		–	
Highest *Moulding:* 1° degree	0.4672	0.018	0.5717	0.002
Highest *Moulding:* 2° degree	1.5347	0.001	1.4396	0.001
Highest *Moulding:* 3° degree	5.5318	0.001	7.2959	0.001
Most severe level of *caput succedaneum:* None	**–**		–	
Most severe level of *caput succedaneum:* Mild	1.4269	0.001	1.2494	0.001
Most severe level of *caput succedaneum:* Moderate/Severe	3.1532	0.001	2.9695	0.001

BMI: Body Mass Index; MHR: Maternal Heart Rate; SBP: Systolic Blood Pressure;

By and large, the performance measures are higher in the maximum score model, followed by the interval models (Table 2). The AUC for the test samples ranged from 78.70 to 93.66% in the full models and 74.73 and 93.72% in the abridged models. The results are similar in the sample training. The sensitivity and specificity values are higher than 65% in all the cases studied, reaching values above 85% in the abridged maximum score model. The accuracy and negative predictive values are greater than 65 and 85%, respectively, for all models. In contrast, the highest positive predictive values observed in the test sample were 53.58 and 58.54%. Additional file 1: Table S4 presents the results of the Hosmer-Lemeshow test to verify the quality of the adjustments. All models have a *p*-value in this test higher than 0.05, which indicates that the models have a good fit to the data.

The variables with greater weight in the increase of the probability of CS of the maximum score model (Table 4) that is, higher estimated positive coefficients are: occurrence of third-degree moulding, moderate/severe *caput succedaneum*, amniotic membrane ruptured with meconium and suspected acute foetal distress. On the other hand, the greatest negative coefficients occurred for: multiparous without previous CS, foetal descent in/below the ischial spines and level of cervical dilatation. Particularly for the obstetric variables used in Robson’s classification [36], the estimated coefficients indicate that the following characteristics are associated with an increased probability of CS: induced labour, multiparous with previous CS, post-term gestational age and cephalic or non-cephalic transverse/posterior foetal presentations. However, the coefficients for multiparous pregnant women without CS and pre-term gestation indicate lower probability of CS.

## Discussion

In this manuscript, we present statistical models for the prediction of the occurrence of CS during labour, using baseline and intrapartum characteristics of 8957 pregnant women and their foetuses in Nigeria and Uganda. We present models obtained from variables recorded during the admission of women to hospitals and throughout labour. Our findings show that it is possible to predict CS and that the performance of this prediction increases considerably when we use information obtained during labour. Among the model versions presented, the maximum score model, which considers the situation of higher risk of women during their care, presented a discriminatory performance higher than 90%.

Our models have a performance equal to or greater than other logistic regression models in the literature, in which the AUC ranged from 70% (16,20–22), 84% [20] and 88% [24]. It should be noted that the previous models used only measures recorded at the hospital admission. Another model has a 75% AUC [25] when also considering information collected during labour. In addition, a study [20] presented AUC close to 94% using regression tree and random forest as an alternative to logistic regression. However, the authors used the hospital in which CS occurred as an independent variable, which made it impossible to apply outside these places.

Comparing the results of the present study with the AUC values ​​observed in the models already proposed [17–24], we observed that the discriminatory capacity of the maximum score model (AUC: 93.66 and 93.72%) is higher. Nevertheless, those of the interval models (2 to 4 h and 4 to 6 h after the start of the active phase) are close to those of the best performance models. On the other hand, our admission and interval models (0 to 2 h) present intermediate discriminatory capacity in relation to what has already been published in the literature. It should be emphasized that the characterization of the samples considered in the models already proposed can limit their application and comparison with the models presented here. This is because, in some cases, the samples are restricted and consider only women with specific characteristics, such as: induced labour [19, 21], spontaneous labour [22], absence of previous CS [21] or previous labour [17, 22], at least one prior CS [23], cephalic presentation [17, 19, 22] and term/post term gestational age [17, 19, 21, 25].

Regarding the practical applicability of the coefficients estimated in each of the models, it can be observed that most of the results are consistent with clinical variables associated with CS already described in the literature. Maternal age was the most used variable in the models already proposed for CS prediction and, as observed in the present study, presents positive coefficients in all studies [17, 18, 20, 22–24], therefore older women present a higher risk of being submitted to CS. Concerning the variables used in the classification of Robson [36], our results are in agreement with other studies that show the increase in the probability of CS for pregnant with induced labour and previous CS [20, 24] and reduction for multiparous women [19, 21, 24]. Regarding gestational age, our models indicated a lower probability of CS for preterm and a higher one for post-term pregnancies when compared to full-term pregnancies. This result contradicts the one observed in the model proposed in 2016 [24], in which pre-term gestation appears positively associated with CS. This difference could have occurred because, in the BOLD project database, preterm pregnancies are older than 34 weeks. Specifically, more than 80% of registered preterm pregnancies are older than 36 weeks, and therefore are associated with obstetric outcomes similar to those of full-term pregnancies.

The models presented here are innovative in relation to what has already been proposed in the literature for using intrapartum variables for CS prediction. This allows the estimation of the probability of CS to be updated throughout labour and not only calculated when women are admitted to hospital. On the other hand, a limitation is the fact that some of the models are somewhat extensive, which can make it difficult to estimate the CS probabilities. To mitigate this problem, each model has an abridged version, with predictive capabilities similar to those of larger models. In addition, the clinical assessments of cervical dilatation were made at irregular intervals in the participating hospitals – few women were rigorously assessed at regular intervals (e.g. every 4 h), which affected negatively the available information for developing interval models. This is because, for the construction of these models, there must be at least one four-centimetre dilatation record, as well as the records of the interest intervals. As many of the intrapartum BOLD records were made according to clinical needs, it was not possible to ensure that all the pregnant women had a four-centimetre dilatation record. In order to reduce the impact of this characteristic of our sample, we developed the maximum score model which was constructed in order to consider all the possible variabilities of the intrapartum measurements, regardless of when they were recorded.

Given the overall discriminatory capacity of the CS prediction models presented in this paper – very good – we hypothesize that such models could be used to support decision making during intrapartum care. The human component should remain as the substantive and final element of decision making during labour and childbirth. However, in certain settings, health care practitioners may lack the necessary experience to make a decision at an optimal timing, and an accurate CS prediction model could function as a decision support tool. Another envisaged application of these models is their integration into obstetric early warning systems, which could reduce delays during intrapartum care. In summary, these models could contribute to maximizing health effects of CS, which can be done by ensuring that all women who need a CS have it, unnecessary CS are avoided and access to safe surgery is strengthened. These goals are particularly important in sub-Saharan Africa, as women who undergo a CS have a greater risk of developing a serious complication or dying [37].

On top of the possible applications at individual level, CS prediction models could be used retrospectively to generate a cluster level predicted CS rate. The average individual predictions of CS risk of an obstetric population equates to the CS risk of that population, which is the same of a predicted CS rate for that population. By comparing the predicted to the observed CS rate, one can identify deviations from what would be expected for that population: CS models could thus be used to benchmark the CS rate of a certain population [24]. Our models were built based on women from Nigeria and Uganda, with single pregnancies, and it is necessary to carry out validation studies for their application in other populations. In addition, the construction of new models based on other populations is also encouraged.

## Conclusion

We conclude that it is possible to predict CS with high accuracy and that intrapartum variables should be considered in this process. Thus, considering the evolution of the computational methods and the improvement in the information infrastructure of maternity settings, we believe that models such as those presented in this work could support clinical decision making and facilitate the analysis of CS rates at the health facility.

## Supplementary information

**Additional file 1: Table S1.** Description of the fixed variables considered in the bivariate analysis step **Table S2.** Description of the dynamic variables considered in the bivariate analysis **Table S3.** Description of variables recorded throughout the labour considered in the univariate analysis **Table S4.** P-value for Hosmer-Lemeshow test in each of the estimated models **Figure S1.** Analysis flow diagram **Figure S2.** ROC curves for models in the training sample (left column) and test sample (right column).

## Data Availability

The datasets used and/or analysed during the current study are available from the corresponding author on reasonable request.

## Abbreviations

AUC: Area Under the Curve
BMI: Body Mass Index
BOLD: Better Outcomes in Labour Difficulty
CI: Confidence Interval
CS: Caesarean Section
DA: Diagnostic Accuracy
DBP: Diastolic Blood Pressure
FHR: Foetal Heart Rate
MHR: Maternal Heart Rate
NPV: Negative Predictive Value
PPV: Positive Predictive value
ROC: Receiver Operate Characteristic
SBP: Systolic Blood Pressure
SE: Sensibility
SP: Specificity.
