# Supplementary material for: Development of caesarean section prediction models: secondary analysis of a prospective cohort study in two sub-Saharan African countries
Source: Reprod Health. 2019 Nov 14;16:165. doi: 10.1186/s12978-019-0832-4 (PMC6854746; doi:10.1186/s12978-019-0832-4)
Supplement: Supplementary file 1 — Additional file 1: Table S1. Description of the fixed variables considered in the bivariate analysis step Table S2. Description of the dynamic variables considered in the bivariate analysis Table S3. Description of variables recorded throughout the labour considered in the univariate analysis Table S4. P-value for Hosmer-Lemeshow test in each of the estimated models Figure S1. Analysis flow diagram Figure S2. ROC curves for models in the training sample (left column) and test sample (right column). [file 12978_2019_832_MOESM1_ESM.docx]

**Supplementary material**

Table S1: Description of the fixed variables considered in the bivariate analysis step.

|  | **Categories/Unit of measurement** |
| --- | --- |
| Maternal height | Centimetres |
| Symphysis-fundal height | Centimetres |
| Foot size (mother) | Centimetres |
| Maternal age | Years |
| BMI by gestational age | Underweight;  Normal*;  Obese;  Overweight |
| Parity and previous caesarean section (CS) | Nulliparous *;  Multiparous with previous caesarean section; Multiparous without previous caesarean section |
| Previous uterine surgery | Yes;  No* |
| Previous abortions | Yes;  No* |
| History of prolonged labour | Yes;  No* |
| Outcome of last pregnancy | No previous pregnancy; Born alive, still alive;  Born alive, deceased;  Stillbirth* |
| Complications with current pregnancy⁺ | Yes;  No* |
| Chronic health conditions prior to pregnancy⁺⁺ | Yes;  No* |
| Gestational age | Pre-term (34 to 36 weeks)  Term (37 to 41 weeks) *  Post-term (42 or more weeks) |
| Mode of onset of labour | Spontaneous*;  Induced |
| Ischial spines prominent | Yes;  No* |
| Cervix position | Anterior*;  Central;  Posterior |
| Foetal movements in the last two hours before admission | No changes or increased* Reduced;  None |

*Indicates the reference category of the variable.

⁺ Placenta praevia, accrete/increta/percreta placenta, abruption placentae, other obstetric haemorrhage, pre-eclampsia, eclampsia, pyelonephritis, malaria, preterm rupture membrane, anaemia, gestational diabetes, other pregnancy complications.

⁺⁺ Chronic hypertension, diabetes mellitus, HIV+, HIV wasting syndrome, chronic anaemia, obesity, heart disease, lung disease, renal disease, other chronic disease

Table S2: Description of the dynamic variables considered in the bivariate analysis

|  | **Categories/Unit of measurement** |
| --- | --- |
| Cervical diltation | Centimetres |
| Maternal heart rate (MHR) | 50 < MHR ≤ 100*;  40 < MHR ≤ 50 or 100 < MHR ≤110; MHR≤40 or MHR>110 |
| Systolic blood pressure (SBP) | 90≤ SBP< 140*;  80≤SBP<90 or 140≤SBP<150  70< SBP<80 or 150≤ SBP<160 SBP≤70 or SBP>=160 |
| Diastolic blood pressure (DBP) | 49< DBP< 90*;  DBP ≤ 49 or 90 ≤ DBP < 100;  100 ≤ DBP < 110;  DBP ≥ 110 |
| Number of uterine contractions in 10 minutes | Less than three;  Three or more* |
| Suspected foetal distress according to foetal heart rate (FHR) | Yes (FHR <120 or FHR>160);  No* |
| Foetal movements | Yes;  No* |
| Foetal station | At/below ischial spines;  Above ischial spines* |
| Foetal presentation | Cephalic anterior*;  Cephalic transverse;  Cephalic posterior;  Other non-cephalic |
| Amniotic membrane status | Intact*;  Ruptured with meconium;  Ruptured without meconium |
| Abnormal axillary temperature (AT) (AT≥37.5 or AT <35.5) | Yes;  No* |
| Time between admission and 4 cm of dilatation greater than 8 hours | Yes;  No*’ |
| Moderate to extreme pain⁺ | Yes;  No* |
| *Moulding* status | None*;  First degree;  Second degree;  Third degree |
| *Caput succedaneum* | None*;  Mild;  Moderate to severe |

*Indicates the reference category of the variable.

*⁺* The BOLD participants were asked to define their labour pain according to a scale with 5 categories: Not at all, Slightly, Moderately, Quite a bit and Extremely.

Table S3: Description of variables recorded throughout the labour considered in the univariate analysis.

|  | **Categories/Unit of measurement** |
| --- | --- |
| Final cervical diltation | Centimetres |
| Most extreme maternal heart rate (MHR) | 50 < MHR ≤ 100*;  40 < MHR ≤ 50 or 100 < MHR ≤110; MHR≤40 or MHR>110 |
| Most extreme systolic blood pressure (SBP) | 90≤ SBP< 140*;  80≤SBP<90 or 140≤SBP<150  70< SBP<80 or 150≤ SBP<160 SBP≤70 or SBP>=160 |
| Most extreme diastolic blood pressure (DBP) | 49< DBP< 90*;  DBP ≤ 49 or 90 ≤ DBP < 100;  100 ≤ DBP < 110;  DBP ≥ 110 |
| Highest number of uterine contractions in 10 minutes | Less than three;  Three or more* |
| At least one suspected foetal distress | Yes (FHR <120 or FHR>160);  No* |
| Presence of foetal movements throughout intrapartum | Yes;  No* |
| Lower foetal station achieved | At/below ischial spines;  Above ischial spines* |
| Final foetal presentation | Cephalic anterior*;  Cephalic transverse;  Cephalic posterior;  Other non-cephalic |
| Final amniotic membrane status | Intact*;  Ruptured with meconium;  Ruptured without meconium |
| Some occurrence of abnormal axillary temperature (AT) (AT≥37.5 or AT <35.5) | Yes;  No* |
| Some occurrence of moderate to extreme pain | Yes;  No* |
| Highest *Moulding* status | None*;  First degree;  Second degree;  Third degree |
| Most severe level of *Caput succedaneum* | None*;  Mild;  Moderate to severe |

*Indicates the reference category of the variable.

⁺ The BOLD participants were asked to define their labour pain according to a scale with 5 categories: Not at all, Slightly, Moderately, Quite a bit and Extremely.

Table S4: P-value for Hosmer-Lemeshow test in each of the estimated models

| **Model** | **P-value** |
| --- | --- |
| Model 1 | 0.4877 |
| Model 2 | 0.2227 |
| Model 2A | 0.3573 |
| Model 3 | 0.3513 |
| Model 3A | 0.6609 |
| Model 4 | 0.5106 |
| Model 4A | 0.1686 |
| Model 5 | 0.8226 |
| Model 5A | 0.7418 |

|  |  |  | |  | | |  | | |  |  |  | |  | |  |  | | |  | | | |  | | |  | | |  | | |  | | | |  | | | | Initial eligible data BOLD  N=9995 | | | | | | | | | | | | | | | | | | | | | | | | | | | | | | | | | | | | | | | | | | | |  | | | |  | | | |  | | | | |  | | | | |  | | |  | |  | | |  | | |  | |  | | |  | | | |  | | | |  | |  | |  | | |  | | |  | | | |  | | | | | |
| --- | --- | --- | --- | --- | --- | --- | --- | --- | --- | --- | --- | --- | --- | --- | --- | --- | --- | --- | --- | --- | --- | --- | --- | --- | --- | --- | --- | --- | --- | --- | --- | --- | --- | --- | --- | --- | --- | --- | --- | --- | --- | --- | --- | --- | --- | --- | --- | --- | --- | --- | --- | --- | --- | --- | --- | --- | --- | --- | --- | --- | --- | --- | --- | --- | --- | --- | --- | --- | --- | --- | --- | --- | --- | --- | --- | --- | --- | --- | --- | --- | --- | --- | --- | --- | --- | --- | --- | --- | --- | --- | --- | --- | --- | --- | --- | --- | --- | --- | --- | --- | --- | --- | --- | --- | --- | --- | --- | --- | --- | --- | --- | --- | --- | --- | --- | --- | --- | --- | --- | --- | --- | --- | --- | --- | --- | --- | --- | --- | --- | --- | --- | --- | --- | --- | --- | --- | --- | --- | --- | --- | --- | --- | --- | --- | --- | --- |
|  |  |  | |  | | |  | | |  |  |  | |  | |  |  | | |  | | | |  | | |  | | |  | | |  | | | |  | | | |  | | |  | | |  | | | |  | | | |  | | |  | | |  | | | |  | | | |  | | |  | | |  | | | |  | | | |  | | | |  | | | |  | | | | |  | | | | | Records with time inconsistencies  n=1038 | | | | | | | | | | | | | | | | | | | | | | | | | | | |  | |  | | |  | | |  | | | |  | | |  |  |  |
|  |  |  | |  | | |  | | |  |  |  | |  | |  |  | | |  | | | |  | | |  | | |  | | |  | | | |  | | | |  | | |  | | |  | | | |  | | | |  | | |  | | |  | | | |  | | | |  | | |  | | |  | | | |  | | | |  | | | |  | | | |  | | | | |  | | | | |  |  |  |  |  |  |  |  |  |  |  |  |  |  |  |  |  |  |  |  |  |  |  |  |  |  |  |  |  | |  | | |  | | |  | | | |  | | |  |  |  |
|  |  |  | |  | | |  | | |  |  |  | |  | |  |  | | |  | | | |  | | |  | | |  | | |  | | | |  | | | | Eligible records for analysis n=8957 | | | | | | | | | | | | | | | | | | | | | | | | | | | | | | | | | | | | | | | | | | | |  | | | |  | | | |  | | | | |  | | | | |  | | |  | |  | | |  | | |  | |  | | |  | | | |  | | | |  | |  | |  | | |  | | |  | | | |  | | | | | |
|  |  |  |  | | |  | | |  | |  |  | |  | |  | |  | | |  | | | |  | | | |  | | |  | | |  | | | |  | | | |  | | |  | | |  | | | |  | | | |  | | |  | | |  | | | |  | | | |  | | |  | | |  | | | |  | | | |  | | | |  | | | |  | | | | |  | | | | |  | | |  | |  | | |  | | |  | |  | | |  | | | |  | | | |  | |  | |  | | |  | | |  | | |  | | | | | | |
|  |  |  |  | | |  | | |  | |  |  | |  | |  | |  | | |  | | | |  | | | |  | | |  | | |  | | | |  | | | |  | | |  | | |  | | | |  | | | |  | | |  | | |  | | | |  | | | |  | | |  | | |  | | | |  | | | |  | | | |  | | | |  | | | | |  | | | | |  | | |  | |  | | |  | | |  | |  | | |  | | | |  | | | |  | |  | |  | | |  | | |  | | |  | | | | | | |
|  |  |  |  | | | **Development Admission Model** | | | | | | | | | | | |  | | |  | | | |  | | | |  | | |  | | |  | | | |  | | | |  | | |  | | | **Development Interval Models** | | | | | | | | | | | | | | | | | | | | | | | | |  | | |  | | | |  | | | |  | | | |  | | | |  | | | | |  | | | | | **Development Maximum Score Model** | | | | | | | | | | | | | | | | | | | | | | | | | | | | | | | | | | | | | | | | | | |  |
|  |  |  |  | | |  | | |  | |  |  | |  | |  | |  | | |  | | | |  | | | |  | | |  | | |  | | | |  | | | |  | | |  | | |  | | | |  | | | |  | | |  | | |  | | | |  | | | |  | | |  | | | Women with no record at 4 cm=6273 | | | | | | | | | | | | | | | | | | | | |  | | | | |  | | |  | |  | | |  | | |  | |  | | |  | | | |  | | | |  | |  | |  | | |  | | |  | | |  | | | | | | |
|  |  |  |  |  |  |  |  |  |  |  |  |  |  |  |  |  |  |  |  |  |  |  |  |  |  |  |  |  |  |  |  |  |  |  |  |  |  |  |  |  |  |  |  |  |  |  |  |  |  |  |  |  |  |  |  |  |  |  |  |  |  |  |  | | | |  | | | |  | | |  | | |  |  |  |  |  |  |  |  |  |  |  |  |  |  |  |  |  |  |  |  |  |  |  |  |  |  |  |  |  |  |  |  |  |  |  | | |  | |  | | |  | | | |  | | | |  | |  | |  | | |  |  |  |  |  |  |  |  |  |  |  |  |  |
|  |  |  |  | | | Available data  n=8440 | | | | | | | | | | | |  | | |  | | | |  | | | |  | | |  | | |  | | | |  | | | |  | | |  | | | Women with some record at 4 cm.  n=2684 | | | | | | | | | | | | | | | | | | | | | | | | |  | | |  | | | |  | | | |  | | | |  | | | |  | | | | |  | | | | | Available data **complete model**  n=8357 | | | | | | | | | | | | | | | |  | | | |  | | | | Available data **reduced model**  n=8640 | | | | | | | | | | | | | | | | | | |  |
|  |  |  |  | | |  | | |  | |  |  | |  | |  |  | |  | | | |  | | | | |  | | |  | | |  | | | |  | | | |  | | |  | | |  | | | |  | | | |  | | |  | | |  | | | |  | | | |  | | |  | | |  | | | |  | | | |  | | | |  | | | |  | | | | |  | | | | |  | | |  | |  | | |  | | |  | |  | | |  | | | |  | | | |  | |  | |  | | |  | | |  | | |  | | | | | |  |  |
|  |  |  |  | | |  | | |  | |  |  | |  | |  |  | |  | | | |  | | | | |  | | |  | | |  | | | |  | | | |  | | |  | | |  | | | |  | | | |  | | |  | | |  | | | |  | | | |  | | |  | | |  | | | |  | | | |  | | | |  | | | |  | | | | |  | | | | |  | | |  | |  | | |  | | |  | |  | | |  | | | |  | | | |  | |  | |  | | |  | | |  | | |  | | | | | |  |  |
|  |  |  | **Training n=5908** | | | | | | | |  |  | | **Test n=2532** | | |  | |  | | | |  | | | | |  | | |  | | |  | | | |  | | | |  | | |  | | |  | | | |  | | | |  | | |  | | |  | | | |  | | | |  | | |  | | |  | | | |  | | | |  | | | |  | | | |  | | | | | **Training n=5850** | | | | | | | | | |  | | |  | | | **Test n=2507** | | | | |  | | | | **Training n=6048** | | | | | | | |  | | |  | | | **Test n=2592** | | | | | | | | |  |  |
|  |  |  |  | | |  | | |  | |  |  | |  | |  |  | |  | | | |  | | | | |  | | |  | | |  | | | |  | | | |  | | |  | | |  | | | |  | | | |  | | |  | | |  | | | |  | | | |  | | |  | | |  | | | |  | | | |  | | | |  | | | |  | | | | |  | | | | |  | | | | |  | | |  | | |  | | | | |  | | | |  | | | |  | | | |  | | |  | | |  | | | | | | | | |  |  |
|  |  |  |  | | |  | | |  | |  |  | |  | |  |  | |  | | | |  | | | | |  | | |  | | |  | | | |  | | | |  | | |  | | |  | | | |  | | | |  | | |  | | |  | | | |  | | | |  | | |  | | |  | | | |  | | | |  | | | |  | | | |  | | | | |  | | | | |  | | |  | |  | | |  | | |  | |  | | |  | | | |  | | | |  | |  | |  | | |  | | |  | | |  | | | | | |  |  |
|  | Women with some record up to 2 hours  n=2683 | | | | | | | | | | | | | | | | | | | | | | | | | | | |  | | |  | | |  | | | |  | | | | Women with some record between 2 and 4 hours  n=1604 | | | | | | | | | | | | | | | | | | | | | | | | | | | | | | | | | | | | | | | | | | |  | | | |  | | | |  | | | | |  | | | | | Women with some record between 4 and 6 hours  n=714 | | | | | | | | | | | | | | | | | | | | | | | | | | | | | | | | | |  | | | | |  | | | |
|  |  |  |  | | |  | | |  | |  |  | |  | |  |  | |  | | | |  | | | | |  | | |  | | |  | | | |  | | | |  | | |  | | |  | | | |  | | | |  | | |  | | |  | | | |  | | | |  | | |  | | |  | | | |  | | | |  | | | |  | | | |  | | | | |  | | | | |  | | |  | |  | | |  | | |  | |  | | |  | | | |  | | | |  | |  | |  | | |  | | |  | | | | | |  | | |  |  |
|  |  |  |  | | |  | | |  | |  |  | |  | |  |  | |  | | | |  | | | | |  | | |  | | |  | | | |  | | | |  | | |  | | |  | | | |  | | | |  | | |  | | |  | | | |  | | | |  | | |  | | |  | | | |  | | | |  | | | |  | | | |  | | | | |  | | | | |  | | |  | |  | | |  | | |  | |  | | |  | | | |  | | | |  | |  | |  | | |  | | |  | | | | | |  | | |  |  |
| Available data **complete model** n=2240 | | | | | | | | | | |  |  | | Available data **reduced model**  n=2302 | | | | | | | | | | | | | | | | |  | | |  | | | | Available data **complete model**  **n**=1170 | | | | | | | | | | | | | | | | | | | | |  | | |  | | | | Available data **reduced model**  n=1233 | | | | | | | | | | | | | | | | | | | | | | | |  | | | |  | | | | | Available data **complete model**  n=460 | | | | | | | | | | | | | | | | | |  | | |  | | | | Available data **reduced model**  **n**=469 | | | | | | | | | | | | | | | | | | |  | | | |
|  |  |  |  | | |  | | |  | |  |  | |  | |  |  | |  | | | |  | | | | |  | | |  | | |  | | | |  | | | |  | | |  | | |  | | | |  | | | |  | | |  | | |  | | | |  | | | |  | | |  | | |  | | | |  | | | |  | | | |  | | | |  | | | | |  | | | | |  | | |  | |  | | |  | | |  | |  | | |  | | | |  | | | |  | |  | |  | | |  | | |  | | | | | |  | | |  |  |
|  |  |  |  | | |  | | |  | |  |  | |  | |  |  | |  | | | |  | | | | |  | | |  | | |  | | | |  | | | |  | | |  | | |  | | | |  | | | |  | | |  | | |  | | | |  | | | |  | | |  | | |  | | | |  | | | |  | | | |  | | | |  | | | | |  | | | | |  | | |  | |  | | |  | | |  | |  | | |  | | | |  | | | |  | |  | |  | | |  | | |  | | | | | |  | | |  |  |
| **Training**  **n=1568** | | | | |  | | | **Test**  **n=672** | | | | |  | | **Training**  **n=1611** | | | | | | |  | | | | **Test**  **n=2302** | | | | | | | | | |  | | | | **Training**  **n=819** | | | | | | | | | |  | | | | **Test**  **n=351** | | | | | | | | | |  | | | | **Training**  **n=863** | | | | | | | | | |  | | | | **Test**  **n=370** | | | | | | | | | | | | |  | | | | | **Training**  **n=322** | | | | | | | | |  | | | **Test**  **n=138** | | | | | | | | |  | | | | **Training**  **n=328** | | | | | | |  | | | **Test**  **n=141** | | | | | | | | | | | |
|  |  |  |  |  |  | | |  |  |  |  |  |  | |  |  |  |  |  |  |  |  | | | |  |  |  |  |  |  |  |  |  |  |  | | | |  |  |  |  |  |  |  |  |  |  |  | | | |  |  |  |  |  |  |  |  |  |  |  | | | |  |  |  |  |  |  |  |  |  |  |  | | | |  |  |  |  |  |  |  |  |  |  |  |  |  |  | | | | |  |  |  |  |  |  |  |  |  |  | | |  |  |  |  |  |  |  |  |  |  | | | |  |  |  |  |  |  |  |  | | |  |  |  |  |  |  |  |  |  |  |  |  |

Figure S1: Analysis flow diagram


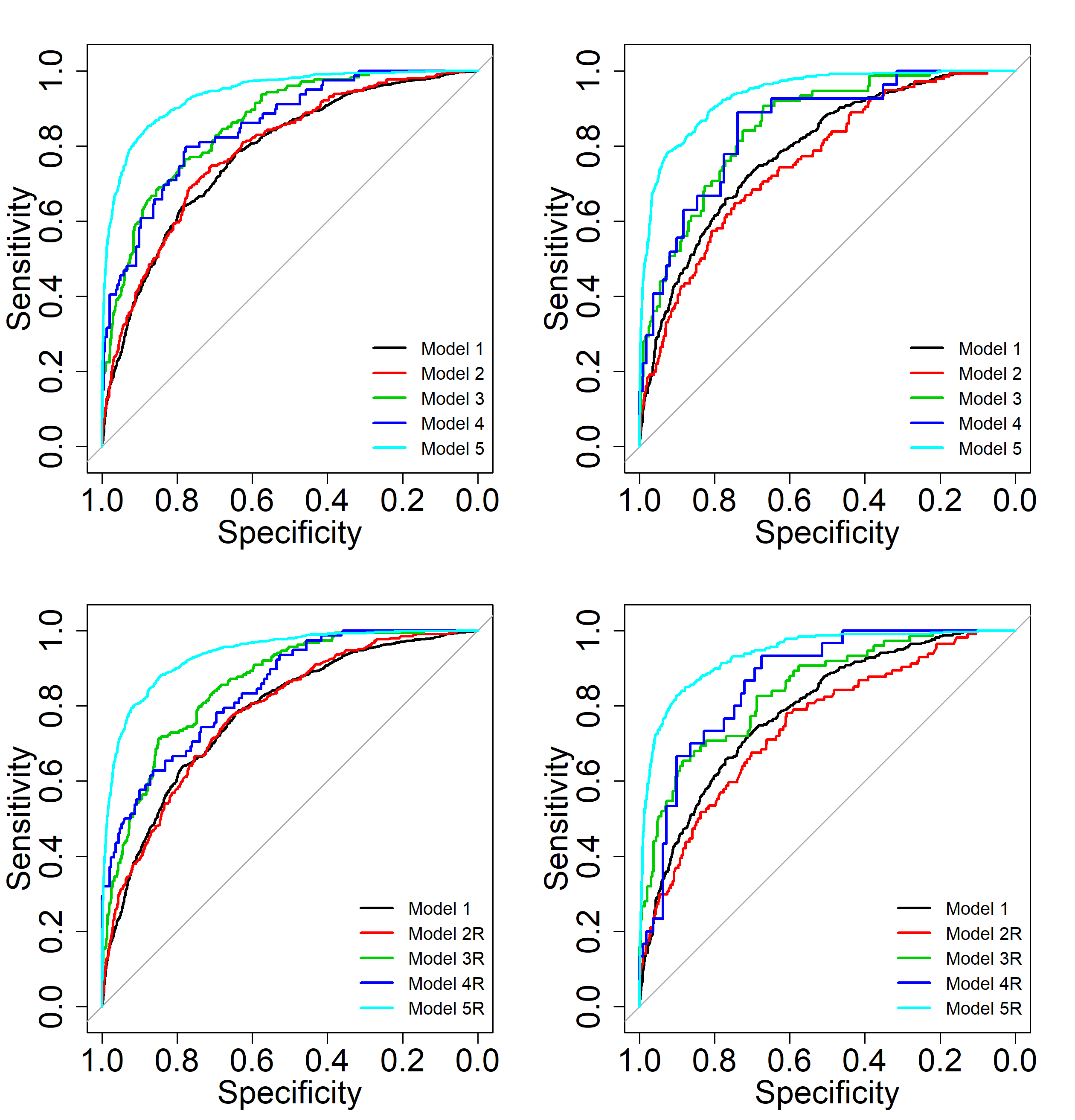


Figure S2: ROC curves for models in the training sample (left column) and test sample (right column)
